# Report of the Surgeon-General of the United States Army, 1887

**Published:** 1888-01

**Authors:** 


### EXTRACTS FROM THE REPORT OF THE SURGEON-GENERAL, UNITED STATES ARMY, 1887.

The money value of the medical and hospital supplies actually issued during the fiscal year ended June 30, 1887, was $159,366.95, and the cost of the supplies required for issue during the current fiscal year will undoubtedly exceed that amount. I base the estimate of the probable cost of the medical and hospital supplies which will be required for issue during the present fiscal year on the fact that the average money value of medical supplies issued annually during the fiscal year July 1, 1876, to June 30, 1887, was approximately $177,515.78, exclusive of all other expenses.

In this connection, I respectfully invite your attention to my estimate of appropriations required by the medical department of the army for the service of the fiscal year ending June 30, 1889, submitted to you on the 15th ultimo, as follows:

For the purchase of medical and hospital supplies, including disinfectants for general post sanitation, expenses of purveying depots, pay of employés, medical care and treatment of officers and enlisted men of the army on duty at posts and stations, for which no other provision is made; for the proper care and treatment of cases in the army suffering from contagious or epidemic diseases; advertising, and other miscellaneous expenses of the medical department (the amount to be expended for pay of employés not to exceed $38,000), $220,000.

For medical and hospital supplies for the army and navy hospital at Hot Springs, Ark., $4,000.

The estimated amounts will, it is believed, be necessary to meet the wants of the medical department for the purposes stated for the ensuing fiscal year. It becomes necessary from time to time to add to the standard supply table new remedies, new instruments, and new standard medical books, and provide them for use by medical officers of the army in the proper diagnosis and treatment of disease.

The limited number of contract surgeons allowed by law necessitates the employment of private physicians, under existing regulations, to furnish medical attendance to officers and enlisted men at stations where there is no medical officer of the army. These physicians are paid by the visit from the "medical and hospital appropriation." Added to this, is the necessary expenditure for the employment of skilled nurses for the proper care and treatment of cases of epidemic and contagious diseases.

I respectfully recommend, as specified in estimate submitted, that the limit of amount of the appropriation to be expended for the pay of employés of the medical department be increased to at least $38,000.

I have the honor to renew my recommendation made in my last annual report that, in order to facilitate the purchase and delivery of medical and hospital supplies, and for the best interests of the service, with a view to economy, Congress be requested to grant authority in the purchase of medical and hospital supplies which cost less than $500 to make such purchases, after due advertisement for bids, without entering into a formal written contract. In many instances, a strict compliance with the letter of the law and existing regulations in preparing the formal executory contracts, five copies of which are required, entails an expense to the government in clerical time and labor fully equal to the cost of the article for which the contract is made. It is not believed that such was the intention of the framers of the law relating to purchases of government supplies.

There were furnished during the year:

In kind:

| | |
|---|---|
| Trusses | 878 |
| Artificial legs | 103 |
| Artificial foot | 1 |
| Artificial arms | 3 |

By commutation:

| | |
|---|---|
| Artificial legs | 301 |
| Artificial feet | 13 |
| Artificial arms | 390 |
| Artificial hands | 6 |
| Apparatus for legs | 343 |
| Apparatus for arms | 517 |

### HEALTH OF THE ARMY FOR THE CALENDAR YEAR ENDING DECEMBER 31, 1886.

On account of its small size, and the nature of the service which it is called upon to perform, the Army of the United States is broken up into small commands, which are scattered over a vast territory comprising regions differing widely in physical features and climatic conditions. In studying the influence exerted upon the health of the troops by peculiarities of elevation, temperature, rain-fall, drainage, etc., it is necessary that the country shall be divided into regions, each of which shall be characterized by physical features and climatic conditions distinct from all others, but uniform, or nearly so, throughout its own area. That division which seems most nearly to meet these requirements, and to be the best adapted to the purposes of this report, is a modification of one made by Mr. Henry Gannett, of the U. S. Geological Survey, and used in the compilation of the mortality and vital statistics of the tenth census, and is shown in the accompanying map. The regions are eleven in number, as follows: The Atlantic Coast, Eastern Timbered Plains and Hills, Appalachian, Northern Lakes, Central Timbered Plains and Hills, Alluvial, Gulf Coast, Prairies, Great Plains, Cordilleras, and Pacific Coast.

On account of the great extent, north and south, of some of these regions, they have, for purposes of comparison, been subdivided into groups of posts.

During the year 1886 no troops were stationed in the Alluvial region, which comprises only the swamp lands of the Mississippi and Red river valleys, and consequently no mention is made of it in the succeeding pages of this report.

Under the head of each region is given a brief description of its distinctive features, and a table showing the military stations within its limits, together with the mean strength of command, admissions to sick report, number constantly non-effective, deaths, and discharges for disability for each station, followed by a general consideration of the subject of the health of the troops serving in the region during the year. Following this are given the principal facts in relation to the health of the whole army; the surgical record for the year; vaccinations; the health of colored troops, of Indian scouts, and of civilian attachés; marriages and births at military stations; the special reports of medical officers; and the hygiene of the army.

### HEALTH OF THE ARMY AS A WHOLE.

The mean strength of the army for the year, including officers and both white and colored enlisted men, was 23,572, as shown by the monthly reports of medical officers. Of this number 21,430 were white, and 2,142 were colored (of African descent). These figures represent the average number present during each day of the year, with commands from which reports were received by the medical department. But the mean strength of the entire army, as shown by the returns of the adjutant-general, was considerably greater than that given above, being 23,737 white and 2,358 colored, or a total of 26,095 men. The discrepancy between the two sets of figures is due to the fact that every officer and enlisted man of the army is accounted for on the returns of the adjutant-general, while the consolidated reports of the medical department show only the strength of the commands from which they are received, and do not include the strength of officers and enlisted men on detached service, and of those commands to which no medical officer is attached, or from which no reports are received.

All the ratios given under the heads of the different regions are based on the mean strength as shown by the medical reports, and, with the exception of the death and discharge rates, are very nearly correct, because no cases of disease or injury are reported for that portion of the army whose mean strength is not reported also, and the number of cases thus lost probably balances the loss in strength. But in the record of deaths and discharges no such loss occurs, for all must, of course, be reported from one source or another; so that these two ratios, as stated for the several regions, are somewhat too high, because they are based upon the mean strength reported by medical officers, which, as above stated, is somewhat less than the actual strength of the respective commands. For the different regions, however, no other strength than that given in the medical reports is at hand, and consequently it has been necessary to use this strength in calculating *all* the ratios for these regions; but for the whole army the mean strength as shown by the adjutant-general's returns is available, and has been used in computing the rates of *death and discharge*. For all other ratios the mean strength shown by the consolidated reports of the medical department is used, and not that of the adjutant-general, which would make these ratios too low, for reasons indicated above.

The Indian scouts employed as auxiliaries to the regular troops averaged for the year 310, but they have not been included in the mean strength of the army or in calculating any of the ratios given in this report for the reason that they have so little in common with the regular troops in respect of their surroundings, habits, manner of living, duration of service, etc., that no comparisons of any value can be made concerning them; and when sick they so rarely come under the observation of medical officers that the reports of commands to which they are attached furnish but little or no information relative to the amount and character of sickness among them.

The rate of constant non-effectiveness was also lowered this year, being 39.4, while that for the previous year was 41.1, and for the preceding decade 44 per 1,000 of mean strength. The average loss of time on account of sickness for each man in the army was 14 days against 15 for 1885, and 16 for the preceding decade.

The death rate was 8.8 per 1,000, being higher than that for 1885, which was 6.9, an exceptionally low figure; but it was, however, considerably lower than the rate for the previous decade, which was 11.4.

### COMPARISON WITH FOREIGN ARMIES.

The following table shows a comparison of the more important ratios bearing upon the health of the Army of the United States with the same ratios for certain foreign armies. In compiling these statistics the latest reports received have been used, and in all cases only the strength actually present with the colors has been considered:

| Army. | Mean strength. | Ratio per 1,000 of mean strength for— Admissions. Hospital or infirmary. | Quarters. | Total. | Deaths. | Discharges for disability. | Constant non-effective force. | Sick-time to each soldier. | Average duration of each case of sickness. |
|---|---|---|---|---|---|---|---|---|---|
| United States (1886) | 23,572 | 546 | 717 | 1,263 | 8.8 | 24.9 | 39.4 | 14 | 11 |
| United States (1876-85, yearly average) | 23,805 | ........ | ........ | 1,672 | 11.4 | 32.2 | 44.0 | 16 | 10 |
| Belgium (1885) | 46,017 | 1,525 | ........ | 1,525 | 5.3 | 10.6 | 30.0 | 11 | 7 |
| Great Britain (1884) | 167,686 | 1,092 | ........ | 1,092 | 8.4 | 18.0 | 56.4 | 21 | 19 |
| France (1878) | 440,614 | 554 | 2,016 | 2,570 | 9.1 | 13.7 | 46.6 | 17 | 7 |
| Italy (1881) | 191,366 | 928 | ........ | 928 | 10.6 | 19.4 | 35.4 | 13 | 14 |
| Prussia and Würtemburg (1881-82) | 353,192 | 324 | 807 | 1,231 | 4.6 | 27.0 | 38.8 | 13 | 11 |

SURGICAL OPERATIONS.

There were 152 surgical operations reported by medical officers during the year; 68 of these were necessitated by injuries, and 84 by various surgical diseases. Antiseptics were used in 71 cases after operations, 21 of which were for shot wounds, and in the treatment of 16 cases of shot wound not operated upon.

The following table shows the number, character, and results of the principal surgical operations performed during the year:

| Nature of Operation. | Disease or injury. | Recovered. | Died. | Total. | Remarks. |
|---|---|---|---|---|---|
| **Removal of tumors** | | | | 21 | |
| Sebaceous cysts | | 7 | | 7 | Scalp, 1; face, 5; neck, 1. |
| Fibroma | | 1 | | 1 | Hand. |
| Lipoma | | 6 | | 6 | Scalp, 1; cheek, 1; side, 1; back, 3. |
| Adenoid | | 1 | | 1 | Neck. |
| Papilloma | | 2 | | 2 | Face, 1; genitals, 1. |
| Epithelioma | | 1 | | 1 | Face. |
| Unclassified | | 3 | | 3 | Face, 2; neck, 1. |
| **Removal of foreign bodies** | | | | 9 | |
| Bullet and shot | Shot wounds | 9 | | 9 | Flesh wounds, 7; fractures, 2. |
| **Opening of abscesses** | | | | 3 | |
| Perityphlitic | Perityphlitis | 1 | 1 | 2 | Incision and drainage. |
| Lumbar | Caries | | 1 | 1 | Incision. |
| **Operations on nerves** | | | | 1 | |
| Stretching of nerves | Neuralgia | 1 | | 1 | Stumps of amputated fingers. |
| **Operations on the eye** | | | | 2 | |
| Iridectomy | Laceration | 1 | | 1 | Lodgment of missile. |
| Excision of the eyeball | ... do | 1 | | 1 | |
| **Operations on the mouth** | | | | 5 | |
| Removal of tonsils | Tonsillitis | 2 | | 2 | |
| Amputation of uvula | Elongated, 2; œdema, 1 | 3 | | 3 | |
| **Operations on arteries** | | | | 1 | |
| Ligation | Incised wound | 1 | | 1 | Ulnar; silk ligature. |
| **Operations on veins** | | | | 2 | |
| For varicocele | Varicocele | 2 | | 2 | |
| **Operations on respiratory organs** | | | | 2 | |
| Paracentesis of pleura | Pleurisy | 2 | | 2 | Aspiration. |
| **Operations on digestive organs** | | | | 17 | |
| For fistula in ano | | 5 | | 5 | Incision. |
| For anal fissure | | 1 | | 1 | Dilation and incision. |
| For hæmorrhoids | | 8 | | 8 | Ligation, 2; excision, 6. |
| Laparotomy | Intestinal obstruction | | 1 | 1 | Death on third day. |
| Paracentesis | Ascites | | 1 | 2 | Not terminated, 1. |
| **Operations on urinary organs** | | | | 8 | |
| Tapping of bladder | Laceration by kick | | 1 | 1 | Aspiration. |
| For stricture of urethra | Traumatic, 1; gonorrheal, 6 | 7 | | 7 | Internal urethrotomy. |
| **Operations on generative organs** | | | | 14 | |
| For phimosis | | 11 | | 11 | Slitting, 2; circumcision, 9. |
| For hydrocele | | 3 | | 3 | Tapping. |
| **Operations on bones** | | | | 8 | |
| Removal of portions of | Necrosis, 5; shot fracture, 1 | 6 | | 6 | Maxilla, 1; clavicle, 1; radius, 1; cranium, 1; tibia, 2. |
| For ununited fractures | Compound fractures | 2 | | 2 | Wiring, 1; splint, 1. |
| **Operations on joints** | | | | 6 | |
| Reduction of dislocations | | 5 | | 5 | Shoulder, 2; elbow, 3. |
| Incisions | Abscess | 1 | | 1 | Elbow joint. |
| **Operations on muscles** | | | | 1 | |
| Myotomy | Anchylosis | 1 | | 1 | Elbow. |
| **Operations on limbs** | | | | 45 | |
| Amputation for injury | | | | 42 | |
| Arm | Shot wound, 1; compound fracture, 1 | 2 | | 2 | Primary, 1; intermediate, 1. |
| Forearm | Shot wound, 2; compound fracture, 1 | 3 | | 3 | Primary, 3. |
| Finger | Shot wound, 12; injury, 12; frost-bite, 4 | 28 | | 28 | Primary, 14; intermediate, 4; secondary, 10. |
| Thigh | Shot wound, 2; compound fracture, 1 | 3 | | 3 | Primary, 1; intermediate, 1; secondary, 1. |
| Leg | Shot wound, 1; compound fracture, 1 | 2 | | 2 | Primary, 1; intermediate, 1. |
| Foot | Frost-bite | 1 | | 2 | Intermediate; double amputation. |
| Toe | Shot wound, 1; compound fracture, 1 | 2 | | 2 | Primary. |
| Amputation for disease | | | | 3 | |
| Finger | Abscess | 1 | | 1 | |
| Thigh | Popliteal aneurism | | 1 | 1 | |
| Toe | Deformity | 1 | | 1 | |
| **Operations on skin, etc** | | | | 7 | |
| Closure of wound | Laceration | 1 | | 1 | Scalp. |
| Ingrown toe-nail | | 6 | | 6 | |
| **Aggregate** | | 144 | 6 | 152 | 1 double amputation; 1 case not terminated. |

HYGIENE OF THE ARMY.

By direction of the Secretary of War, on July 15, 1885, paragraph 2315 of the Army Regulations was amended to read as follows :

An important part of the duty of a medical officer of the army is the supervision, under the direction of his immediate commander, of the hygiene of the post or command to which he is attached, and the recommendation of such measures as he may deem necessary to prevent or diminish disease among the troops. For this purpose he shall at least once a month examine and note in the medical history of the post the sanitary condition of the quarters, including all buildings belonging to the post, the character and cooking of the rations, the amount and quality of the water supply, the drainage, and the clothing and the habits of the men, and make a report thereon in writing to the commanding officer, with such recommendations as he may deem proper. If the recommendations be approved and carried out, the medical officer shall note the fact in the medical history of the post. If the action recommended be deemed impracticable or undesirable, the commanding officer shall indorse his objections on the report and forward it to the department commander. A copy of such indorsement shall be furnished to the medical officer, who shall record it in the medical history of the post. A copy of each report, and of the action of the commanding officer thereon, will be forwarded as soon as practicable, through the usual military channels, to the surgeon-general for his information.

The good effect of this regulation is clearly shown by the character of the sanitary reports received from medical officers during the year 1886. Some of these reports, it is true, are merely perfunctory and contain little or no information which is of interest or value, but the majority of them bear evidence of careful and painstaking investigation on the part of the officers making them, and a full appreciation of the great responsibility devolving upon them in connection with the performance of this portion of their duty.

As is to be expected, out of the large number of recommendations made on the great variety of subjects dealt with in the monthly sanitary reports, a few were clearly not practicable, and concerning a few others, differences of opinion arose between the commanding officer and the medical officer ; but by far the greater portion of the sanitary measures recommended were carefully considered, practical, and capable of being easily carried into effect with the means at command, and it is gratifying to note, the medical officers as a rule received the cordial support and co-operation of their immediate commanders in their efforts to increase the health and comfort of the commands to which they were attached. That these efforts have been attended with a large measure of success is evidenced by the diminished rates of sickness and mortality in the army for the year 1886, as compared with previous years.

Of all the unsanitary conditions reported during the year, that which appears to have been most serious, as well as most widely spread, was found to exist in connection with the privies, sinks, urinals, and other similar conveniences provided for the use of the troops. The cess-pool and privy-vault, or pit, is universally condemned as being a nuisance under any circumstances, and frequently a source of the gravest danger to health, and that they should be tolerated at so many of our military stations, in spite of the well known evils inseparably connected with their use, and in disregard of the repeated warnings and remonstrances of medical officers, is inexplicable upon any other ground than that the mere matter of convenience has been allowed to outweigh all other considerations. At some of the older posts, the privies, especially those of the officers' quarters, have for years been erected over sinks dug in the earth and moved from time to time, as these pits became unpleasantly full, to other locations close by, new pits being dug and the old ones covered over with earth, until, in the language of several of the reports on this subject, the back yards have become literally "honey-combed with deposits of filth." It seems to be urgently demanded that steps should be taken not only to do away with the evils complained of, but to prevent their recurrence, by the official prohibition of the use at military stations of such methods as are known to be dangerous in the disposal of excrementitious matters and waste of various kinds, and by the adoption, instead, of such system, or systems, as will meet as nearly as possible the differing requirements of different localities, and of modern sanitary science as well.

The lack of proper bathing facilities, not only for enlisted men but for officers, is seriously felt at many posts, and is a subject which well deserves greater attention than it has heretofore received.

FOOD.

With the few exceptions noted below, the food supplied to the troops was reported to be sufficient in quantity, of excellent quality, and usually well cooked. The opinion seems to be quite prevalent among both medical and line officers, serving at southern posts, that bacon should be issued instead of salt pork, for the reason that in those localities the latter is hardly ever eaten by the men, and the saving can only be sold at a very low price, if at all; so that the food value to the soldier of this portion of the ration is greatly diminished. On the other hand, bacon is occasionally relished by the men even in the hottest weather, and that which is not consumed by them always finds a ready sale at a good price, thus augmenting the company or hospital fund, and allowing the purchase of fresh vegetables and other articles of diet not included in the ration; so that the soldier suffers no loss in his food supply, whatever may be the pecuniary loss to the government through the greater shrinkage in weight, and liability to spoil, of bacon over pork.

BATHING FACILITIES.

In many, if not in most, of the older barracks and quarters the provision made for purposes of personal cleanliness of the occupants, in the way of properly constructed bath-rooms and lavatories, is notoriously inadequate and unsatisfactory, and, in the construction of some of the more modern buildings as well, this most important feature has either not been given the prominence which it deserves, or has been neglected altogether. In but very few of the sanitary reports of medical officers is the statement made that the bathing facilities are adequate and properly constructed, while in many reports, particularly those of western posts, reiterated complaints are made of deficiencies in these respects.

ARMY MEDICAL MUSEUM.

Twenty-one hundred and twenty-three specimens were added to the museum during the year; an increase of 768 over the number added the preceding year. This makes the total number of specimens in the museum June 30, 26,072, classified as follows:

| | Specimens. |
|---|---|
| Pathological Section: | |
| In museum June 30, 1886 | 9,583 |
| Received during the year | 255 |
| In museum June 30, 1887 | 9,838 |
| Section of Comparative Anatomy: | |
| In museum June 30, 1886 | 1,622 |
| Received during the year | 52 |
| In museum June 30, 1887 | 1,674 |
| Anatomical Section: | |
| In museum June 30, 1886 | 2,345 |
| Received during the year | 607 |
| In museum June 30, 1887 | 2,952 |
| Microscopical Section: | |
| In museum June 30, 1886 | 9,510 |
| Received during the year | 536 |
| In museum June 30, 1887 | 10,046 |
| Miscellaneous Section: | |
| In museum June 30, 1886 | 346 |
| Received during the year | 438 |
| In museum June 30, 1887 | 784 |
| Provisional Section: | |
| Pathological specimens in museum June 30, 1886 | 270 |
| Received during the year | 173 |
| Pathological specimens in museum June 30, 1887 | 443 |
| Anatomical specimens in museum June 30, 1886 | 273 |
| Received during the year | 62 |
| Anatomical specimens in museum, June 30, 1887 | 335 |

The more interesting specimens added to the museum collection during the year ending June 30, 1887, were a series of plaster casts and bony preparations collected by the late Professor F. H. Hamilton, of New York; models of the heart and vessels of various fishes, mussels, etc., prepared by Auzoux, of Paris; skeletons and crania of ancient Laps and Finns; a series of corroded metal injection specimens of the vessels of the kidney, liver and lung, and a series of old microscopes illustrating the gradual improvement in these instruments during the last two centuries.

With the removal of the museum to the new fireproof building will uudoubtedly come large accessions of specimens contributed by physicians in all parts of the United States.

The recommendation made in the last annual report that authority be granted by Congress for the publication of an illustrated catalogue of the museum is respectfully renewed. A large part of the manuscript for such a catalogue has been prepared and it will form a work in three large volumes, which will be of great value and interest to the medical profession and will be of great benefit to the museum itself.

### LIBRARY.

The following table shows the additions made to the library during the fiscal year:

| Description. | On hand June 30, 1886. | Added during fiscal year. | Total, June 30, 1887. |
|---|---|---|---|
| Medical journals | 24,116 | 1,261 | 25,377 |
| Medical transactions | 3,532 | 117 | 3,649 |
| Bound theses | 1,385 | .......... | 1,385 |
| Bound pamphlets | 1,331 | 231 | 1,562 |
| Other medical books | 46,368 | 6,824 | 53,192 |
| Total | 76,732 | 8,433 | 85,165 |
| Medical theses | 42,212 | 3,067 | 45,279 |
| Medical pamphlets | 64,419 | 9,955 | 74,374 |
| Total | 106,631 | 13,022 | 119,653 |

There were presented to the library during the year, 375 books and 5,683 pamphlets.

There is urgent need of a large amount of binding for the library, there being now 7,400 volumes unbound, many of which are recent works which are almost daily called for by readers, but which should not be used until they are bound. This accumulation of unbound books is due to the fact that the amount allowed for binding for this office has been insufficient for several years.

### INDEX-CATALOGUE.

Volume VIII of the Index-Catalogue, including from "Legier" to "Medicine (Naval)," forming a volume of 1,078 pages, has been printed and the edition distributed to those institutions and persons who have received the previous volumes. The usefulness of this work to the medical writers and teachers, not only of the United States but of other countries, can hardly be overestimated, and it is very desirable that it should be completed as rapidly as possible. The preparation of the manuscript of Volume IX is well advanced, and the first part of it is now going to press.

### THE NEW MUSEUM AND LIBRARY BUILDING.

I take great pleasure in reporting that the new building to contain the museum, library, and hospital records, for which there has been such urgent need, is now rapidly approaching completion. It is thoroughly fireproof, and gives space for arranging the books, records and specimens in a way which will add largely to their utility. A portion of the building has been turned over to this department, and the most important part of the pension records and of the library have been moved to it and thus made secure from fire.

In this connection I desire to express my appreciation of the care and labor bestowed in the supervision of the construction of this building by the officers who have had charge of it, viz., Colonel T. L.

Casey and Colonel J. M. Wilson, of the U. S. Engineer Corps. They have had many difficulties, but it is believed that the result is as satisfactory as the too limited appropriation at their disposal for this work would permit.

### HOSPITAL CORPS.

"An act to organize the Hospital Corps of the Army of the United States" having been approved by the President, a board of competent and experienced officers was detailed to carry out its purpose and prepare the necessary plans for its working details; the report of the board having been approved by the Secretary of War and promulgated in orders to the army, the necessary transfers to the corps of enlisted men as acting hospital stewards and privates are being made as rapidly as possible.

The conditions of transfer are such that the corps will consist of a body of intelligent men, who, under instruction from the medical officers of the army in the important duties of caring for the sick and attendance upon the wounded, will furnish an organization under the control of the medical department, which will enable it to meet promptly all the accidents of peace and emergencies of war.

A system of lectures upon, and practical demonstration in, first aid to the wounded has already been successfully introduced, and drills for stretcher and ambulance service will soon be perfected.

The onerous and frequently dangerous service performed by the privates of the corps entitles them, in my opinion, to an extra pay. I accordingly recommend that an allowance of 20 cents per day be added to their present pay.

### ARMY AND NAVY GENERAL HOSPITAL, HOT SPRINGS, ARK.

This hospital was opened for the reception of patients January 17, 1887, under charge of Major R. S. Vickery, surgeon U. S. Army.

| | | |
|---|---|---|
| Admitted as patients from January 17 to June 30, 1887 | | 45 |
| Returned to sick leave | 4 | |
| Left hospital | 2 | |
| Returned to company | 8 | |
| Returned to duty—commissioned officers | 2 | |
| Returned to furlough | 1 | |
| Died | 1 | |
| | | 18 |
| Remaining in hospital June 30, 1887 | | 27 |

The total number of applications from commissioned officers and enlisted men for admission is fifty-three, of which five were rejected.

The number of patients in the hospital July 1, 1887, was—

| | |
|---|---|
| Officers of the army | 5 |
| Officers of the navy | 1 |
| Enlisted men of the army | 21 |
| Total | 27 |

Total number of beds for patients, July 1, 1887:

| | |
|---|---|
| For officers | 16 |
| For enlisted men | 64 |

It is believed that the benefits to the army and navy from the use of the waters of this justly celebrated place will be great, and liberality in the supply of funds for its maintenance and improvement is earnestly recommended.

### MISCELLANEOUS.

The Army Medical Board convened in New York city on the 6th of April, 1885, was dissolved by order from the War Department dated April 9, 1887. The following is a recapitulation of the work performed by the board during its session:

| | |
|---|---|
| Assistant surgeons examined for promotion | 8 |
| Candidates for appointment in the medical corps invited to appear for examination | 93 |
| Candidates found qualified | 16 |
| Candidates rejected | 21 |
| Candidates who withdrew after partial examination | 32 |
| Total examined | 69 |

| | |
|---|---|
| Candidates who failed to appear for examination | 19 |
| Candidates who declined to appear for examination | 5 |
| Number invited but not examined | 24 |

Lieutenant-Colonel John Moore, assistant medical purveyor, was appointed surgeon-general, with the rank of brigadier-general, November 18, 1886, *vice* Murray, retired from active service.

There are no vacancies in the medical corps of the army.

The number of medical officers permanently disabled is becoming a matter of serious embarrassment to the efficiency of this department and renders necessary the employment, under contract, of private physicians; an expensive and unsatisfactory procedure, to remedy which it is hoped that Congress may be induced to take action by special or general legislation.

With this view I urgently recommend that an increase of twenty assistant surgeons be authorized; which addition, it is believed, will meet the necessities of the service.

